# Unilateral lower limb cellulitis-like edema as an atypical cutaneous manifestation of antisynthetase syndrome: a case report and targeted literature review

**DOI:** 10.3389/fmed.2026.1942019

**Published:** 2026-09-09

**Authors:** Yue Zhang, Jia Su, Ling Zhong, Lan Shang, Huiying Wan

**Affiliations:** 1 Department of Dermatology, Sichuan Provincial People’s Hospital, School of Medicine, University of Electronic Science and Technology of China, Chengdu, China; 2 School of Medicine and Life Science, Chengdu University of Traditional Chinese Medicine, Chengdu, China; 3 Department of Radiology, Sichuan Provincial People’s Hospital, University of Electronic Science and Technology of China, Chengdu, China

**Keywords:** antisynthetase syndrome, cellulitis-like rash, cutaneous edema, inflammatory myopathy, interstitial lung disease

## Abstract

**Background:**

Antisynthetase syndrome (ASyS) is a distinct subtype of idiopathic inflammatory myopathy (IIM), characterized by multisystem involvement, including the lungs, skin, skeletal muscles, and joints, as well as the presence of anti-aminoacyl-tRNA synthetase (ARS) antibodies. While 8% to 30% of patients present with classic dermatomyositis-like rashes, atypical skin involvement can also occur. Rarely, patients may develop cutaneous edema that mimics cellulitis, which is highly susceptible to misdiagnosis.

**Case presentation:**

A 72-year-old woman presented with a 1-week history of erythema, swelling, and pain in the right lower limb. Physical examination showed marked edema with a peau d’orange appearance, local warmth, and tenderness. Given the detection of fungal hyphae on the right foot, cellulitis was initially suspected. However, the lesions did not improve after 5 days of cefuroxime sodium treatment, and she developed right gastrocnemius tenderness, markedly elevated muscle enzymes, and interstitial changes on chest computed tomography. Myositis antibody testing was positive for anti-melanoma differentiation-associated gene 5(anti-MDA5) and anti-histidyl-tRNA synthetase(anti-Jo-1) antibodies. Right gastrocnemius biopsy showed fiber-size variation and scattered endomysial inflammatory infiltration, without necrosis or perifascicular atrophy; immunohistochemistry showed focal/patchy major histocompatibility complex class I (MHC-I) expression and weak non-perifascicular sarcoplasmic myxovirus resistance protein A (MxA) positivity. Antisynthetase syndrome was diagnosed. Moderate-dose oral prednisone was added and cefuroxime sodium was discontinued. Eight days later, erythema, swelling, and myalgia improved, with marked decreases in muscle enzymes and hypersensitive C-reactive protein(hs-CRP). After discharge, mycophenolate mofetil(MMF) and nintedanib were added. Three months later, the skin lesions had completely resolved, creatine kinase, lactate dehydrogenase, high-sensitivity troponin, alanine aminotransferase (ALT), and aspartate aminotransferase (AST) nearly normalized; interstitial lung disease (ILD) improved, and pleural/pericardial effusions markedly decreased.

**Discussion:**

Notably, this case highlights that ASyS may mimic infectious cellulitis and cause misdiagnosis. Inflammatory myopathy should be considered when atypical skin lesions are accompanied by elevated muscle enzymes and ILD.

## Introduction

Antisynthetase syndrome (ASyS) is a distinct subtype of idiopathic inflammatory myopathy (IIM) characterized by the presence of anti-aminoacyl-tRNA synthetase (ARS) antibodies (1). Its clinical features include myositis, interstitial lung disease (ILD), non-erosive arthritis, Raynaud's phenomenon, mechanic's hands, and fever. Cutaneous manifestations of ASyS are heterogeneous. Approximately 8%–30% of patients develop typical dermatomyositis-like rashes, such as Gottron's rash, heliotrope rash, and the V-sign (2, 3). However, atypical or absent skin involvement may also occur. Cutaneous edema presenting as erythematous, cellulitis-like swelling is an exceptionally uncommon manifestation of ASyS and other idiopathic inflammatory myopathies, including dermatomyositis, frequently leading to misdiagnosis. Pathophysiologically, this presentation is considered an extension of severe muscle edema and capillary leakage (4), the latter of which is driven by increased vascular permeability secondary to the loss of the endothelial junctional protein VE-cadherin. Additionally, cutaneous edema is increasingly recognized as a marker of severe disease, which often warrants early and aggressive therapy (5).

Therefore, in patients with atypical cutaneous findings that show poor response to antibiotics, particularly when accompanied by elevated muscle enzymes or interstitial lung abnormalities, an underlying autoimmune inflammatory process should be considered. In this report, we aimed to describe a rare case of ASyS presenting initially with cellulitis-like cutaneous edema, integrate previous literature and summarize the relevant clinical features and diagnostic challenges, and highlight the potential importance of early recognition to facilitate timely diagnosis and management.

## Case presentation

**Figure 1:**
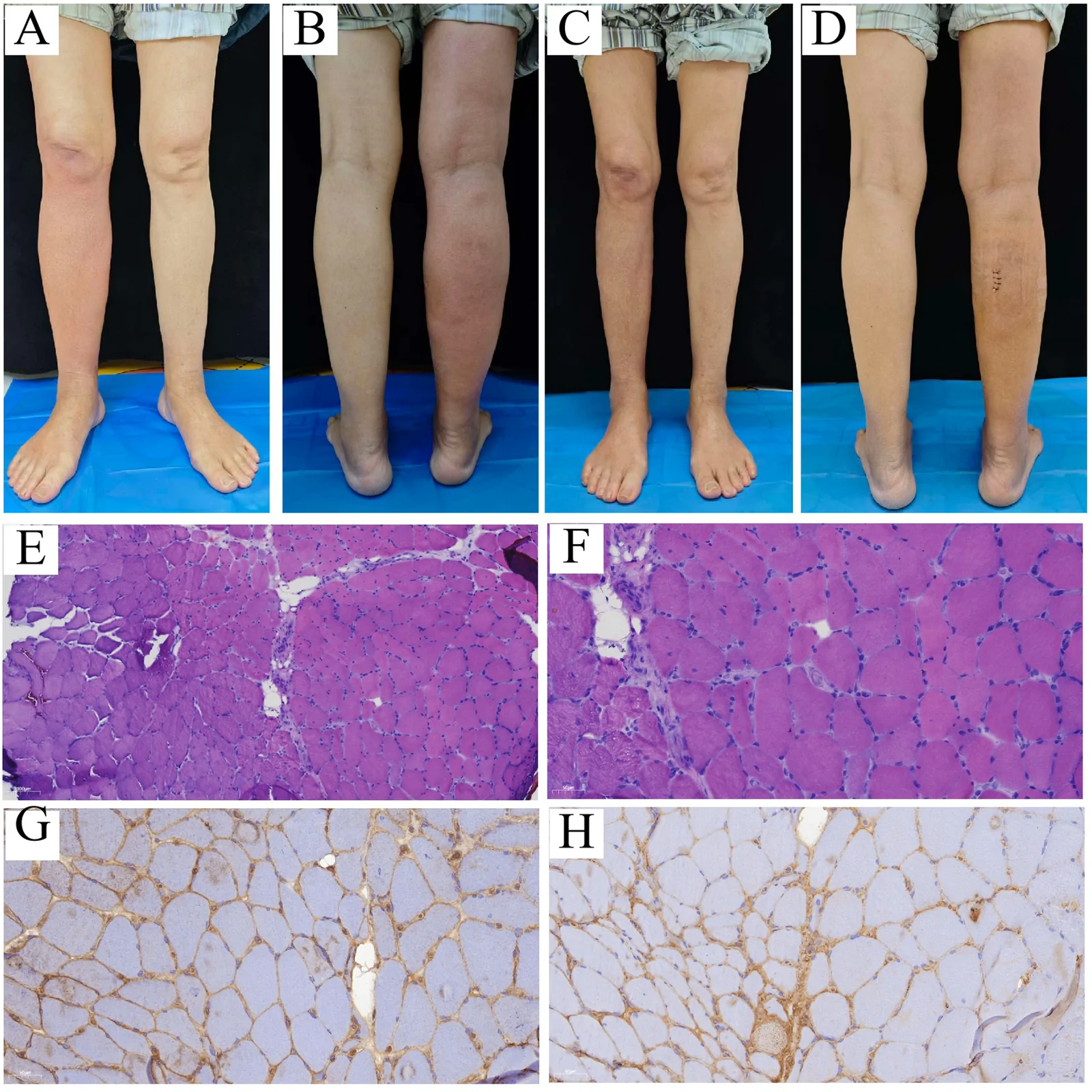
Clinical and histopathological analyses. **(A,B)** Physical examination revealed diffuse erythema and edema with peau d’orange-like appearance involving the right lower limb. **(C,D)** Marked improvement of erythema and edema after oral prednisone therapy for 8 days. **(E)** Hematoxylin and eosin (H&E) staining of the right gastrocnemius muscle biopsy showing variation in fiber size and scattered lymphocytic and plasma cell infiltration within the endomysium, without necrosis, regeneration, or phagocytosis, and absence of perifascicular atrophy (original magnification, 100×). **(F)** Higher magnification of H&E staining of the muscle biopsy (original magnification, 200×). **(G)** Immunohistochemical staining showing focal or patchy expression of major histocompatibility complex class I (MHC-I), (original magnification, 200×). **(H)** Myxovirus resistance protein A (MxA) staining showing weak sarcoplasmic positivity with a non-perifascicular distribution pattern (original magnification, 200×).

A 72-year-old Chinese woman presented with a 1-week history of erythema, swelling, and pain in the right lower extremity. The lesion initially appeared over the right knee and gradually extended to the entire right lower leg. Physical examination showed diffuse erythema and swelling with poorly defined margins. The skin was warm, tense, and mildly tender, with a peau d'orange appearance, but without ulceration, exudation, or purulence (Figures 1A,B). No rash was found elsewhere, and she had no fever, arthralgia, or Raynaud's phenomenon. Fungal hyphae were detected in the interdigital spaces of the right foot. Laboratory tests showed elevated hs-CRP (37.8 mg/L; reference range, 0–5 mg/L), erythrocyte sedimentation rate(ESR, 58 mm/h; 0–20), ALT (42 U/L; 7–40), AST (78 U/L; 13–35), lactate dehydrogenase (LDH, 622 U/L; 120–250), and ferritin (1,453 ng/mL; 4.63–204), with decreased hemoglobin (92 g/L; 115–150), albumin (27.3 g/L; 40–55), and complement C3 (0.787 g/L; 0.9–1.8). The antinuclear antibody (ANA) test revealed a cytoplasmic-granular pattern (1:100) and a nuclear-granular pattern (1:100). White blood cell, neutrophil, and eosinophil counts, procalcitonin(PCT), creatinine, B-type natriuretic peptide (BNP), coagulation parameters and D-dimer were within normal limits. Routine urinalysis was negative for both proteinuria and hematuria. Tests for antineutrophil cytoplasmic antibodies (PR3-ANCA and MPO-ANCA) were all negative. Doppler ultrasonography demonstrated patent deep veins with normal compressibility and flow, with no evidence of deep venous thrombosis. Based on the above findings, cellulitis was initially suspected.

**Figure 2:**
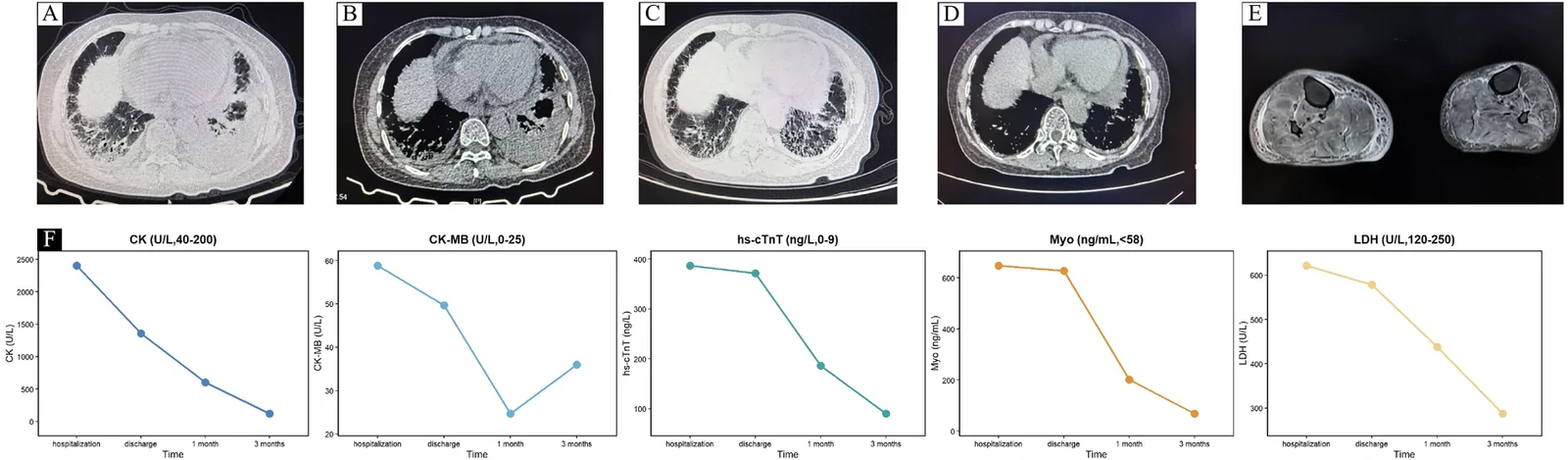
Radiological findings and serial changes in serum enzyme levels during the clinical course. **(A,B)** Baseline non-contrast chest CT scans. Lung window **(A)** reveals bilateral interstitial lung changes consistent with NSIP, characterized by scattered reticular and patchy opacities, ground-glass opacities predominantly involving the bilateral lower lobes, with partial consolidation, atelectasis, traction bronchiectasis, and focal pleural thickening; mediastinal window **(B)** demonstrates moderate pleural effusion and pericardial effusion.**(C,D)**: Follow-up non-contrast chest CT after 3 months of treatment, demonstrating improvement of the interstitial lung disease and a significant reduction in both pleural and pericardial effusions. **(E)** Non-contrast magnetic resonance imaging of the lower legs showing soft tissue swelling and hyperintense signals in the calf muscles on T2-weighted fat-suppressed images. **(F)** Serial changes in serum creatine kinase (CK), creatine kinase-myocardial band (CK-MB), high-sensitivity cardiac troponin T (hs-cTnT), myoglobin (MYO), and lactate dehydrogenase (LDH) levels.

However, the skin lesions did not improve after 5 days of intravenous cefuroxime sodium (1.5 g every 8 h), and the patient developed pain in the right gastrocnemius muscle. Creatine kinase (CK, 2,403 U/L;40–200), CK-MB activity (creatine kinase-MB isoenzyme activity, 58.8 U/L;0–25), high-sensitivity troponin (hs-cTnT, 387 ng/L;0–9), and myoglobin (648 ng/mL; <58) increased sharply, while hs-CRP rose to 45.64 mg/L. Chest computed tomography (CT) showed nonspecific interstitial pneumonia, with small pleural and pericardial effusions (Figures 2A,B). Holter monitoring revealed sinus rhythm with a normal electrical axis, whereas echocardiography showed no ventricular dysfunction.

Further history revealed previous thyroid surgery. Three months earlier, she had been diagnosed with hypothyroidism and hyperlipidemia and had started levothyroxine 25 μg daily and rosuvastatin 10 mg daily. Elevated liver enzymes had also been detected 3 months earlier, for which she received oral hepatoprotective therapy. One month before presentation, she had intermittently experienced exertional chest tightness and dyspnea, accompanied by reduced grip strength in both hands.

A myositis antibody panel showed positivity for anti-Jo-1, anti-MDA5, anti-Ku, anti-Ro-52, and anti-PM-Scl 100 antibodies (by immunoblot); whereas anti-3-hydroxy-3-methylglutarylcoenzyme A reductase (anti-HMGCR) and anti-signal recognition particle (anti-SRP) antibodies were negative. Thyroid function tests showed reduced thyroid-stimulating hormone (TSH) and free triiodothyronine (FT3) compared with three months earlier (TSH, 9.48 vs. 16 mIU/L; reference range, 0.27–4.20 mIU/L; FT3, 2.6 vs. 3.6 pmol/L; reference range, 3.1–6.8 pmol/L). free thyroxine (FT4) was normal (18.2 pmol/L; 12.0–22.0), and both thyroid peroxidase antibody (TPOAb) and thyroglobulin antibody (TgAb) were negative. Magnetic resonance imaging (MRI) of the right lower leg demonstrated soft-tissue swelling and hyperintense signals within the lower-leg muscles on fat-suppressed T2-weighted images, consistent with myositis (Figure 2E). Electromyography indicated peripheral neuropathy in both lower limbs, more pronounced on the right side. To further clarify the diagnosis, we performed a muscle biopsy following MRI-guided targeted localization of active lesions. Right gastrocnemius biopsy showed variation in muscle fiber size and scattered endomysial lymphocytic and plasma-cell infiltration, without necrosis, regeneration, phagocytosis, or perifascicular atrophy (Figures 1E,F). Immunohistochemistry showed focal/patchy MHC-I expression and weak sarcoplasmic MxA positivity in a non-perifascicular pattern (Figures 1G,H). Based on the clinical and pathological findings, antisynthetase syndrome was ultimately diagnosed.

## Treatment and follow-up

To exclude statin-associated myopathy, rosuvastatin was replaced with ezetimibe. Oral prednisone was added at an initial dose of 30 mg daily, and cefuroxime sodium was discontinued. Eight days later, erythema and swelling subsided, myalgia improved, and muscle enzyme, cardiac biomarkers and hs-CRP levels decreased markedly (Figures 1C,D). After discharge, the patient was followed up in the rheumatology department. Prednisone was gradually tapered to 20 mg one week later, followed by a 2.5 mg dose reduction every 4 weeks thereafter. Following multidisciplinary evaluation, MMF was initiated 0.5 g twice daily for long-term immunosuppression, while nintedanib 100 mg twice daily was added as an adjunctive antifibrotic therapy because of the presence of interstitial lung involvement and potential risk factors for fibrotic progression. Three months later, the prednisone dose was reduced to 12.5 mg daily. Meanwhile, CK, LDH, hs-cTnT, ALT, and AST levels had largely normalized (Figure 2F), interstitial lung disease had improved, with a significant reduction in pleural and pericardial effusions (Figures 2C,D), and the skin lesions had completely resolved. Chest tightness, dyspnea, hand weakness, and myalgia also improved. Regular oral levothyroxine was maintained, and improvements in thyroid function were observed over the subsequent course (Supplementary Table S1). No serious infectious complications or treatment-related adverse events occurred during follow-up.

## Discussion

ASyS is defined by the presence of ARS antibodies and predominantly affects middle-aged and older women. Its classic triad comprises myositis, ILD, and arthritis, although the disease often begins with a single manifestation, most commonly ILD (1). Less frequent features include pleural effusion, pericardial effusion, myocarditis, pulmonary hypertension, and dysphagia. When myositis is present, patients typically develop proximal muscle weakness and myalgia, usually more prominent in the lower than in the upper limbs (6). In patients with ASyS-associated ILD, approximately half have an insidious onset with progressive dyspnea and cough, whereas 8.9% develop rapidly progressive ILD, a severe and potentially life-threatening phenotype (7). Serologically, Multiple anti-ARS antibodies have been identified to date. Anti-Jo-1 is the most common, and its level correlates positively with CK levels and disease activity (8). Muscle magnetic resonance imaging can support the diagnosis of inflammatory myopathy and guide biopsy site selection. In the present case, anti-Jo-1 positivity, inflammatory myopathy, and ILD fulfilled both the 2010 Connors criteria and the 2011 Solomon criteria (9, 10)(The diagnosis of IIM is based on the latest EULAR/ACR classification criteria (11, 12), and patients can be classified as “definite IIM”. Details are provided in the Supplementary Material). The patient also scored 68.6 according to the 2025 CLASS project criteria (Supplementary Material), meeting the classification threshold for ASyS (13).

The major diagnostic challenge was the cellulitis-like presentation. Tinea pedis is a common predisposing factor for erysipelas and cellulitis, and this patient had erythema, warmth, pain, and cutaneous edema, leading to the initial misdiagnosis. However, she had no fever, leukocytosis, neutrophilia, or procalcitonin elevation, and MRI showed muscle involvement rather than isolated cutaneous or subcutaneous edema. Lack of response to antibiotics and rapid improvement with glucocorticoids supported an autoimmune inflammatory process. Additionally, other infectious and non-infectious causes of unilateral lower-limb swelling should also be considered. Infectious diseases include abscess and necrotizing soft tissue infection, whereas non-infectious mimics include focal inflammatory myositis, fasciitis, vasculitis, Wells syndrome, localized lymphedema, panniculitis, venous obstruction, chronic venous insufficiency, trauma, ruptured Baker's cyst, and malignancy. In this case, the absence of fever, leukocytosis, neutrophilia, elevated PCT, purulent lesions, or imaging evidence of abscess or vascular thrombosis argued against major infectious causes. Furthermore, the lack of purpura, ulcers, necrosis, subcutaneous nodules, eosinophilia, coagulation abnormalities, elevated D-dimer, proteinuria, hematuria, and ANCA positivity made vasculitis, Wells syndrome, panniculitis, and venous obstruction less likely. The acute onset, proximal-to-distal progression of edema, marked elevation of muscle enzymes, positive myositis-associated antibodies, and rapid response to immunosuppressive therapy were also inconsistent with primary lymphedema or chronic venous disorders. Although focal inflammatory myositis and fasciitis can present with unilateral swelling, MRI in this patient demonstrated inflammatory involvement of the lower-leg muscles without a dominant focal lesion pattern or significant fascial abnormalities. Meanwhile, marked muscle enzyme elevation, anti-Jo-1 antibody positivity, and concomitant ILD supported a systemic autoimmune inflammatory myopathy rather than an isolated localized process.

ASyS should be distinguished from dermatomyositis. According to Bohan/Peter criteria, characteristic rashes such as Gottron's rash, heliotrope rash, and the V-sign (14), are important features supporting the diagnosis of dermatomyositis, none of which were present in this patient. Histopathologically, classic dermatomyositis is characterized by perifascicular atrophy, perivascular or perimysial inflammatory infiltrates predominantly composed of B cells and CD4 + T lymphocytes, and sarcoplasmic MxA positivity with a perifascicular distribution pattern (15, 16). In contrast, pathological features of ASyS are variable. Muscle biopsies may present with dermatomyositis-like changes, such as perifascicular atrophy and perivascular inflammatory infiltration, or nonspecific findings, including myofiber degeneration, scattered endomysial and perivascular inflammatory infiltrates, or isolated upregulation of MHC-I expression on the sarcolemma (17). Here, MHC-I positivity supported IIM; but absence of perifascicular atrophy and weak, non-perifascicular MxA expression represented a nonspecific changes, which were inconsistent with typical dermatomyositis pattern. Ryan et al. reported that approximately two-thirds of patients with ASyS lack characteristic dermatomyositis rashes, whereas extramuscular manifestations, including ILD, arthritis, and myocarditis, are more prominent (15). Although anti-MDA5 was positive, the overall clinical, serologic, and pathological findings favored ASyS rather than dermatomyositis. Notably, anti-MDA5 antibodies are confirmed to be associated with rapidly progressive interstitial lung disease (RP-ILD). A previous study reported that all dermatomyositis patients with concomitant anti-MDA5 and anti-ARS antibodies developed ILD, and those with dual-antibody positivity had significantly higher peak ferritin levels than patients positive for anti-ARS antibodies alone (18). In our patient, both anti-MDA5 and anti-ARS antibodies were detected, with ILD identified on chest CT and markedly elevated ferritin levels (>1,000 ng/mL). However, with early diagnosis and appropriate treatment, no pulmonary symptom progression or radiological deterioration was observed during follow-up. The coexistence of anti-MDA5 with other autoantibodies can result in highly atypical phenotypes. For example, Sama et al. (19) reported a case of dual anti-SAE1 and anti-MDA5 positivity presenting with classic cutaneous signs and significant biopsy-proven inflammatory myopathy, yet featuring entirely normal creatine kinase (CK) levels. In contrast, our patient exhibited dual anti-Jo-1 and anti-MDA5 positivity accompanied by marked CK elevation and unilateral cellulitis-like edema, further demonstrating the extreme clinical and biochemical heterogeneity of anti-MDA5-associated overlap syndromes.

In addition to anti-Jo-1 and anti-MDA5 antibodies, this patient tested positive for anti-Ku, anti-Ro-52, and anti-PM-Scl 100 antibodies, representing a rare penta-positive myositis autoantibody profile. This complex serological pattern posed diagnostic challenges regarding a potential overlap syndrome. Anti-Ku, anti-Ro-52, and anti-PM-Scl100 are classified as myositis-associated antibodies (MAAs), which may indicate overlap features with other connective tissue diseases. Anti-Ro-52 antibodies commonly coexist with ARS antibodies. Previous studies have shown that anti-Ro-52 most frequently co-occurs with anti-Jo-1 antibodies in predominantly Caucasian cohorts, whereas its coexistence with anti-PL 7, anti-PL 12, or anti-EJ antibodies is more commonly reported in Southeast Asian populations; additionally, anti-Ro-52 positivity is also associated with a higher prevalence and increased severity of ILD (20). Anti-Ku and anti-PM-Scl 100 antibodies are typically associated with myositis/systemic sclerosis overlap syndromes and have been linked to poorer treatment responses (21). However, the patient did not fulfill the 2013 ACR/EULAR classification criteria for systemic sclerosis due to the absence of other systemic sclerosis features, including skin thickening of the fingers, abnormal nailfold capillaries, Raynaud's phenomenon, or anti-centromere antibody positivity (36). Despite the complex autoantibody profile suggesting a possible overlap phenotype, the overall clinical, serological, and pathological findings favored a primary diagnosis of ASyS rather than overlap syndrome.

Hypothyroid myopathy was also considered but was unlikely to be the primary diagnosis. Although hypothyroidism may contribute to muscle symptoms or elevated muscle enzyme levels, it cannot fully account for the patient's clinical presentation. Her hypothyroidism was postsurgical rather than autoimmune, thyroid autoantibodies were negative, and thyroid function improved with stable levothyroxine, whereas myopathy improved only after glucocorticoid therapy. Moreover, hypothyroid myopathy is not associated with anti-ARS antibodies or ILD, and histologically usually shows myxoid degeneration and muscle atrophy rather than inflammatory infiltration (22). Statin exposure may have contributed to muscle injury in this patient; therefore, statin-associated muscle symptoms (SAMS) and statin-associated autoimmune myopathy (SAAM) cannot be completely excluded. Nevertheless, several findings argue against these diagnoses. Typical SAMS usually develop within 4–8 weeks of treatment, present as bilateral symmetrical myalgia, improve after withdrawal, and rarely cause marked enzyme elevation (23). In contrast, the patient presented with unilateral myalgia and significantly elevated muscle enzymes 3 months after starting rosuvastatin, which was inconsistent with typical SAMS. SAAM is an immune-mediated necrotizing myopathy characterized by anti-HMGCR antibodies (24). In this patient, anti-HMGCR and anti-SRP antibodies were negative, and biopsy showed no necrotizing myopathy.

**Figure 3:**
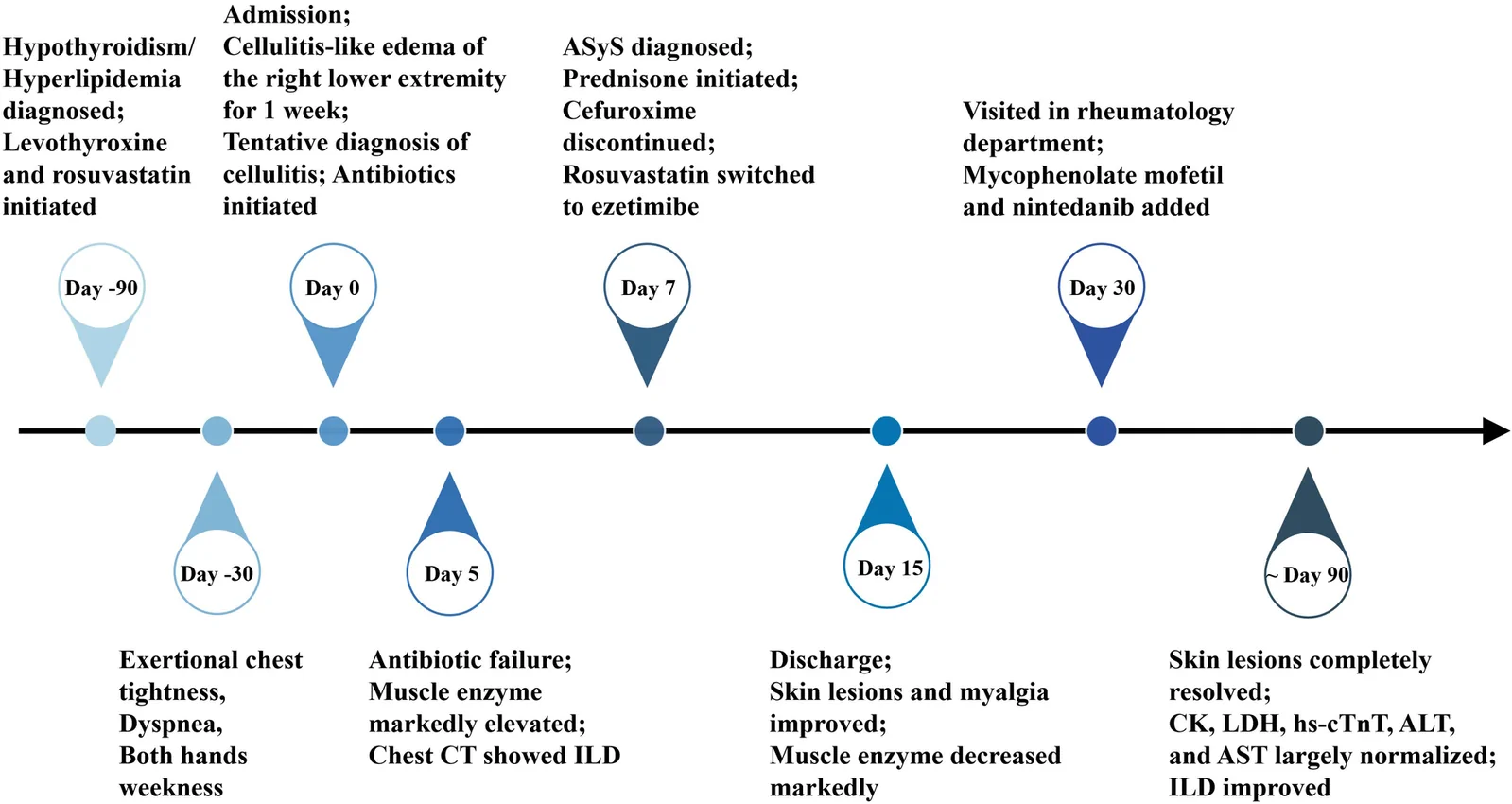
Chronological timeline of the clinical course, diagnosis, treatment, and follow-up.

Using “antisynthetase syndrome” and “edema” as major search terms, we searched in PubMed for English-language case reports published from January 2006 to April 2026. Studies were included if they reported confirmed ASyS patients with edema-related manifestations and provided sufficient clinical information. Eligible study designs included case reports and case series because of the rarity of ASyS-associated edema. Studies were excluded if they were duplicates, reviews, non-clinical studies, reports without confirmed ASyS diagnosis, or cases without edema-related manifestations. The detailed search strategy is provided in Supplementary Material. Initial search identified 61 articles. Two investigators independently screened the titles and abstracts, and 42 articles were subsequently excluded. Ultimately, 10 previously reported cases in 9 articles of ASyS-associated edema were identified and compared with the present case. We mainly extracted data of age, sex, disease duration, clinical manifestations, edema location, serological parameters, antibody findings, treatment regimens, and clinical outcomes, with the findings summarized in Table 1. Among the previous cases, cutaneous edema in ASyS appears more common in young to middle-aged women with disease duration ranging from 9 days to 6 months. Edemamay involve the eyelids (32), unilateral or bilateral extremities (25, 27, 29–31, 33), or present as anasarca (26, 28), although localized peripheral edema is more commonly observed. In two patients, edema was more likely secondary to fluid retention caused by cardiac involvement. In contrast, the majority of reported cases, including the present patient, exhibited features suggesting that edema represented a primary inflammatory manifestation of ASyS. Previous studies have proposed “edematous dermatomyositis” as a distinct clinical subtype, which is often associated with higher disease activity and poorer outcomes (34, 35). Similarly, edema may represent a rare but characteristic clinical phenotype of ASyS. Nevertheless, given the limited number of reported cases, whether edematous ASyS constitutes a distinct clinical subtype requires validation in larger clinical studies. Cutaneous manifestations may include typical dermatomyositis-like rashes (25, 31, 32), nonspecific cellulitis-like edema (29), or absence of a reported rash (26–28, 30). Respiratory symptoms, particularly dyspnea, represent the most frequent initial presentation. Commonly reported associated manifestations include ILD, polymyositis, and myocarditis, with ILD being the most frequently reported. Nonspecific interstitial pneumonia (NSIP) is the predominant radiological pattern observed in ILD patients (25, 31–33). Serologically, autoantibody profiles vary among cases, although anti-Jo-1 and anti-Ro-52 antibodies are the most frequently detected. Treatment usually involves glucocorticoids combined with immunosuppressants, such as MMF or methotrexate. Refractory cases may require multi-drug regimens or biologic therapies, including rituximab (25, 33). Outcomes are generally favorable, with most patients achieving substantial improvement after appropriate treatment. In our patient, skin lesions and ILD improved after treatment with combined prednisone, MMF, and nintedanib, highlighting the potential importance of early diagnosis and multidisciplinary management (Figure 3). Nintedanib was added as an adjunctive antifibrotic therapy despite the absence of respiratory deterioration. Although evidence from randomized trials is lacking, emerging studies suggest potential benefits of nintedanib in ASyS-ILD, particularly when combined with glucocorticoids and immunosuppressive agents (20). Further studies are warranted to clarify its role in this population.

**Table 1:** Summary of case reports on antisynthetase syndrome with cutaneous edema.

Case	Author/Year	Age/Sex	Duration	Initial Presentation	Edema Site	Skin Manifestation	Comorbidities	Elevated enzymes	Antibodies	ILD Type	Maintenance Therapy	Outcome
1	Hrishikesh S. Kulkarni (25)	43/M	NA	Dyspnea	Bilateral lower extremity	Scaly hands, thickened skin over MCP joints	ILD, PM, Myocarditis	cTnI	Jo-1, Ro-52	NSIP	Prednisone + MMF + Rituximab	Improvement
2	Aadil M. Khan (26)	18/F	2.5 months	Proximal muscle weakness, myalgia, dysphagia, dysphonia, dyspnea, anasarca	Anasarca	—	PM	CK, LDH	Jo-1, Ro-52, Mi-2β, ANA-HEp2	—	Methylprednisolone + MTX	50% improvement in muscle weakness, partial bulbar weakness
3	Yinying Li (27)	69/F	0.5 months	Coughing	Bilateral lower extremity	—	ILD	No	Ha, MDA5	NA	Prednisone + Nintedanib + Tacrolimus	Improvement
4	Jiaqing Xiong (28)	70+/F	2 months	Anasarca, exertional dyspnea	Anasarca	—	Hypertension, Hyperlipidemia, Hypothyroidism	cTnT, CK, CK-MB	Jo-1, Ro-52, PM/Scl-100	OP	Prednisolone + MMF	Improvement
5	Jia Liu (29)	12/F	9 days	Fever	Left lower extremity	Light purple rash on extensor elbows	No	CK	Ro-52, PL-7	NA	Prednisone + MTX	Improvement
6	Mikael Ebbo (30)	33/M	2 weeks	Myalgia, Distal pitting edema	Extremities	—	Not mentioned	CK	Jo-1	NA	Steroids + MTX	Improvement
7	Mikael Ebbo (30)	43/F	6 months	Edema and symmetrical pain of lower limbs and forearms	Extremities	—	ILD	CK	Jo-1, Ro-52	NA	Steroids + MTX	Improvement
8	Karim Asi (31)	15/M	6 weeks	Polyarthralgias, fever, bilateral eye and foot swelling	Bilateral eye, foot	Heliotrope rash	JDM, ILD, Coronary artery dilation	CK	PL-12	NSIP	Prednisone + Cyclosporine + MTX + Aspirin, then monthly cyclophosphamide IV	Improvement
9	Ellen De Langhe (32)	44/F	—	Progressive dyspnea, cough, swelling of fingers and eyelids, Raynaud's phenomenon	Fingers, eyelids	Heliotrope rash, mechanic's hands	ILD, Polyarthritis, Myocarditis	CK	PL-7	NSIP	Methylprednisolone + MMF	Improvement
10	Miroslawa M. Gorecka (33)	57/M	1 month	Dyspnea	Extremities	NA	Learning disability, ILD, Myocarditis	cTnT, CK-MM	Jo-1	NSIP	Oral steroids + Rituximab	Improvement
11	Present case	72/F	1 week	Lower-limb erythema, swelling and pain.	Right lower extremity	Cellulitis-like rash on right lower extremity	ILD, Hypothyroidism, Hyperlipidemia	CK, CK-MB, cTnT, Mb	Jo-1, Ro-52, PM/Scl-100, MDA5, Ku	NSIP	Prednisone + MMF + nintedanib	Improvement

Cases were searched through PubMed for English-language case reports and case series published from January 2006 to April 2026. Two investigators independently screened studies using predefined criteria. The table includes all eligible cases identified but is not intended to be an exhaustive review of the literature. ASyS, antisynthetase syndrome; ILD, interstitial lung disease; PM, polymyositis; JDM, juvenile dermatomyositis; MMF, mycophenolate mofetil; MTX, methotrexate; NSIP, nonspecific interstitial pneumonia; OP, organizing pneumonia; MCP, metacarpophalangeal; CK, creatine kinase; CK-MB, creatine kinase-myocardial band; LDH, lactate dehydrogenase; cTnI, cardiac troponin I; cTnT, cardiac troponin T; Mb, myoglobin; ANA, antinuclear antibody; HEp-2, human epithelial type 2; NA, not applicable.

## Limitations

Several limitations should be acknowledged. First, the complex serological profile and pathological findings were not entirely typical. Initial chemiluminescence-based ENA assay (ENA-CIA) testing reported results as antibody index (AI, a manufacturer-defined relative index, with negative <10.0), identifying positive anti-Jo-1 (AI 26.2) and anti-Ro-52 (AI 89.3). Subsequent qualitative immunoblotting detected a quintuple-positive profile (anti-Jo-1, MDA5, Ku, Ro-52, PM-Scl100); however, this was not confirmed by immunoprecipitation. Therefore, potential assay-related cross-reactivity or false positive results cannot be completely excluded. In addition, muscle biopsy lacked typical perifascicular atrophy or necrosis and showed only weak, non-perifascicular MxA expression, which is atypical for both ASyS and dermatomyositis. This may be attributable to biopsy performed several days after glucocorticoid initiation, which could have partially attenuated inflammatory changes. Second, cardiac involvement was not fully assessed because cardiac MRI, coronary evaluation, and cTnI testing were not performed. Although holter and echocardiography were unremarkable, subclinical myocarditis or coronary artery disease could not be completely excluded. Pulmonary function tests were also unavailable due to poor patient cooperation, limiting the assessment of lung function impairment. Third, the therapeutic regimen, including MMF combined with nintedanib, represented an individualized multidisciplinary decision rather than a guideline-based first-line strategy. The optimal glucocorticoid dose and combination therapy for ASyS remain uncertain. Fourth, longer-term follow-up is required to further assess pulmonary outcomes, sustained treatment response, and potential risk of disease progression or relapse. Finally, as a single-case report with a targeted literature review, this study is subject to small sample size and referral bias. Larger studies are required to validate the clinical significance of cutaneous edema as a rare manifestation of ASyS.

## Conclusion

This case illustrates an uncommon presentation of ASyS with unilateral cellulitis-like edema as the initial manifestation. In patients with atypical cellulitis unresponsive to antibiotics, the presence of elevated muscle enzymes or interstitial lung abnormalities should prompt consideration of an underlying inflammatory myopathy. Awareness of this presentation may facilitate earlier diagnosis and appropriate management of antisynthetase syndrome.

## Data Availability

The original contributions presented in the study are included in the article/Supplementary Material, further inquiries can be directed to the corresponding author/s.

## References

[1] Cavagna L Trallero-Araguás E Meloni F Cavazzana I Rojas-Serrano J Feist E. Influence of antisynthetase antibodies specificities on antisynthetase syndrome clinical Spectrum time course. J Clin Med. (2019) 8(11):2013. 10.3390/jcm8112013 31752231 PMC6912490

[2] Wu S Xiao X Zhang Y Zhang X Wang G Peng Q. Novel endotypes of antisynthetase syndrome identified independent of anti-aminoacyl transfer RNA synthetase antibody specificity that improve prognostic stratification. Ann Rheum Dis. (2024) 83(6):775–86. 10.1136/ard-2023-225284 38395605

[3] Lilleker JB Vencovsky J Wang G Wedderburn LR Diederichsen LP Schmidt J. The EuroMyositis registry: an international collaborative tool to facilitate myositis research. Ann Rheum Dis. (2018) 77(1):30–9. 10.1136/annrheumdis-2017-211868 28855174 PMC5754739

[4] Anantharajah A Vucic S Tarafdar S Vongsuvanh R Wilcken N Swaminathan S. Prominent subcutaneous oedema as a masquerading symptom of an underlying inflammatory myopathy. Intern Med J. (2017) 47(2):217–21. 10.1111/imj.13324 28201858

[5] Li W Sun K Huang J Han X Shen Y Xu X. Updates on endothelial injury in dermatomyositis: clinical correlations and potential mechanisms. J Invest Dermatol. (2026). 10.1016/j.jid.2026.06.1269 42470402

[6] Noguchi E Uruha A Suzuki S Hamanaka K Ohnuki Y Tsugawa J. Skeletal muscle involvement in antisynthetase syndrome. JAMA Neurol. (2017) 74(8):992–9. 10.1001/jamaneurol.2017.0934 28586844 PMC5710328

[7] Marco JL Collins BF. Clinical manifestations and treatment of antisynthetase syndrome. Best Pract Res Clin Rheumatol. (2020) 34(4):101503. 10.1016/j.berh.2020.101503 32284267

[8] Arcani R Rey L Mazziotto A Bertin D Kaplanski G Jarrot P-A. Anti-Jo-1 autoantibodies: biomarkers of severity and evolution of the disease in antisynthetase syndrome. Arthritis Res Ther. (2023) 25(1):125. 10.1186/s13075-023-03116-5 37481643 PMC10362709

[9] Connors GR Christopher-Stine L Oddis CV Danoff SK. Interstitial lung disease associated with the idiopathic inflammatory myopathies: what progress has been made in the past 35 years? Chest. (2010) 138(6):1464–74. 10.1378/chest.10-0180 21138882

[10] Solomon J Swigris JJ Brown KK. Myositis-related interstitial lung disease and antisynthetase syndrome. J Bras Pneumol. (2011) 37(1):100–9. 10.1590/S1806-37132011000100015 21390438 PMC3676869

[11] Bottai M Tjärnlund A Santoni G Werth VP Pilkington C De Visser M. EULAR/ACR classification criteria for adult and juvenile idiopathic inflammatory myopathies and their major subgroups: a methodology report. RMD open. (2017) 3(2):e000507. 10.1136/rmdopen-2017-000507 29177080 PMC5687535

[12] Lundberg IE Tjärnlund A Bottai M Werth VP Pilkington C De Visser M. 2017 European league against rheumatism/American college of rheumatology classification criteria for adult and juvenile idiopathic inflammatory myopathies and their Major subgroups. Arthritis Rheumatology. (2017) 69(12):2271–82. 10.1002/art.40320 29106061 PMC5846474

[13] Faghihi-Kashani S Yoshida A Bozan F Zanframundo G Rozza D Loganathan A. Clinical characteristics of anti-synthetase syndrome: analysis from the classification criteria for anti-synthetase syndrome project. Arthritis Rheumatology. (2025) 77(4):477–89. 10.1002/art.43038 39467037 PMC11936500

[14] Bohan A Peter JB. Polymyositis and dermatomyositis (first of two parts). N Engl J Med. (1975) 292(7):344–7. 10.1056/NEJM197502132920706 1090839

[15] Hum RM Lilleker JB Lamb JA Oldroyd AGS Wang G Wedderburn LR. Comparison of clinical features between patients with anti-synthetase syndrome and dermatomyositis: results from the MYONET registry. Rheumatology. (2024) 63(8):2093–100. 10.1093/rheumatology/kead481 37698987 PMC11292049

[16] Waisayarat J Wongsuwan P Tuntiseranee K Waisayarat P Dejthevaporn C Khongkhatithum C. Sarcoplasmic myxovirus resistance protein A: a study of expression in idiopathic inflammatory myopathy. J Inflamm Res. (2023) 16:5417–26. 10.2147/JIR.S433239 38026261 PMC10676103

[17] Tian XL Liu HY Zhang LN Zhang Y Li W Liu Q. Pathological characteristics of skeletal muscle in anti-synthase syndrome patients. Chin J Rheumatol. (2020) 24(9):597–603. 10.3760/cma.j.c141217-20190711-00254

[18] Chen X Zhang L Jin Q Lu X Lei J Peng Q. The clinical features and prognoses of anti-MDA5 and anti-aminoacyl-tRNA synthetase antibody double-positive dermatomyositis patients. Front Immunol. (2022) 13:987841. 10.3389/fimmu.2022.987841 36110863 PMC9468482

[19] Sama S Rasheed N Shen K Khanlou N Han K Rodriguez-Pla A. Atypical presentation of anti-small ubiquitin-like modifier 1 and melanoma differentiation-associated gene 5 antibody positive dermatomyositis presenting with significant inflammatory myopathy on biopsy and normal creatine kinase levels: a case report. SAGE Open Med Case Rep. (2024) 12:2050313X241298862. 10.1177/2050313X241298862 39539645 PMC11558725

[20] Patel P Marinock JM Ajmeri A Brent LH. A review of antisynthetase syndrome-associated interstitial lung disease. Int J Mol Sci. (2024) 25(8):4453. 10.3390/ijms25084453 38674039 PMC11050089

[21] Schlecht N Sunderkötter C Niehaus S Nashan D. Update on dermatomyositis in adults. J Dtsch Dermatol Ges. (2020) 18(9):995–1013. 10.1111/ddg.14267 32985813

[22] Qiang F He Q Wang L Sheng J. Polymyositis-like hypothyroid myopathy: diagnostic challenges and therapeutic outcomes in a case series. Clin Exp Med. (2025) 25(1):286. 10.1007/s10238-025-01828-3 40788546 PMC12339587

[23] Warden BA Guyton JR Kovacs AC Durham JA Jones LK Dixon DL. Assessment and management of statin-associated muscle symptoms (SAMS): a clinical perspective from the national lipid association. J Clin Lipidol. (2023) 17(1):19–39. 10.1016/j.jacl.2022.09.001 36115813

[24] Tiniakou E. Statin-Associated autoimmune myopathy: current perspectives. Ther Clin Risk Manag. (2020) 16:483–92. 10.2147/TCRM.S197941 32581543 PMC7266943

[25] Kulkarni HS Gutierrez FR Despotovic V Russell TD. A 43-year-old man with antisynthetase syndrome presenting with acute worsening of dyspnea. Chest. (2015) 147(6):e215–e9. 10.1378/chest.14-2402 26033135

[26] Khan AM Ahmad F Rehman U Jindal H Harimohan H. A clinical case of polymyositis complicated by antisynthetase syndrome. Cureus. (2021) 13(1):e12737. 10.7759/cureus.12737 33614339 PMC7883527

[27] Li Y Ke J Huang S Lin S Lei S Huang H. Acute exacerbation of anti-ha antibody-positive antisynthetase syndrome-associated interstitial lung disease: a case report. Front Immunol. (2025) 16:1614777. 10.3389/fimmu.2025.1614777 40574857 PMC12197957

[28] Xiong J Balakrishnan T Fong W. Anasarca as the first presentation of anti-synthetase syndrome. BMJ Case Rep. (2024) 17(5):e258359. 10.1136/bcr-2023-258359 38749521

[29] Liu J Nie N Zhang R Wang D Lin Y Chang H. Anti-synthetase syndrome with anti-PL-7 antibody positive in a child: a case report and literature review. Front Immunol. (2025) 16:1525432. 10.3389/fimmu.2025.1525432 40098963 PMC11911354

[30] Ebbo M Chagnaud C Figarella-Branger D Legall S Harle JR Schleinitz N. Antisynthethase syndrome presenting as peripheral limb fasciitis. Joint Bone Spine. (2013) 80(5):528–30. 10.1016/j.jbspin.2013.02.013 23618805

[31] Asi K Gourishankar A Kamdar A. Coronary artery dilation associated with anti-synthetase syndrome in an adolescent. Pediatr Rheumatol Online J. (2019) 17(1):3. 10.1186/s12969-019-0304-y 30630507 PMC6329119

[32] De Langhe E Lenaerts J Bossuyt X Westhovens R Wuyts WA. Mechanic’s hands in a woman with undifferentiated connective tissue disease and interstitial lung disease–anti-PL7 positive antisynthetase syndrome: a case report. J Med Case Rep. (2015) 9:82. 10.1186/s13256-015-0571-2 25888844 PMC4407325

[33] Gorecka MM Tummon O Smyth Y O'Regan A. When your immune system falls out with your heart: an important lesson on antisynthetase myocarditis. BMJ Case Rep. (2018) 2018:bcr-2018-226019. 10.1136/bcr-2018-226019 30158272 PMC6119403

[34] Milisenda JC Doti PI Prieto-González S Grau JM. Dermatomyositis presenting with severe subcutaneous edema: five additional cases and review of the literature. Semin Arthritis Rheum. (2014) 44(2):228–33. 10.1016/j.semarthrit.2014.04.004 24830790

[35] Xu X Huang J Wang X Li M Yang J. Pseudoangioedema in dermatomyositis patients indicates severe disease and poor prognosis. J Am Acad Dermatol. (2022) 86(2):474–5. 10.1016/j.jaad.2021.09.043 34592381

[36] van Den Hoogen F Khanna D Fransen J Johnson SR Baron M Tyndall A. 2013 Classification criteria for systemic sclerosis: an American college of rheumatology/European league against rheumatism collaborative initiative. Arthritis Rheum. (2013) 65(11):2737–47. 10.1002/art.38098 24122180 PMC3930146

