# Supplementary material for: Unilateral lower limb cellulitis-like edema as an atypical cutaneous manifestation of antisynthetase syndrome: a case report and targeted literature review
Source: Front Med (Lausanne). 2026 Sep 9;13:1942019. doi: 10.3389/fmed.2026.1942019 (PMC13597253; doi:10.3389/fmed.2026.1942019)
Supplement: Supplementary file 1 [file Supplementaryfile1.docx]

| **TableS1.** **Serial laboratory findings aligned with the clinical timeline** | | | | | |
| --- | --- | --- | --- | --- | --- |
| **Parameter** | **Reference range** | **Day 0** | **Day 5** | **Day 15** | **~Day 90** |
| hs-CRP | 0-5 mg/L | 37.8 | 45.64 | 7.65 | 6.11 |
| ESR | 0-20 mm/h | 58 | - | - | 14 |
| PCT | 0-0.05 ng/mL | 0.04 | - | - | - |
| Ferritin | 4.63-204 ng/mL | 1453 | - | - | - |
| WBC | 3.50-9.50×10^^9^/L | 6.14 | 8.49 | 8.15 | 9.08 |
| Neutrophils | 1.80-6.30×10^^9^/L | 4.22 | 6.04 | 5.26 | 5.12 |
| Eosinophils | 0.02-0.52×10^^9^/L | 0.16 | 0.16 | 0.11 | 0.12 |
| Hb | 115-150 g/L | 92 | 104 | 105 | 137 |
| CK | 40-200 U/L | - | 2403 | 1359 | 121 |
| CK-MB activity | 0-25 U/L | - | 58.8 | 49.7 | 36 |
| Myo | <58 ng/mL | - | 648 | 628 | 68 |
| hs-cTnT | 0-9 ng/L | - | 387 | 372 | 90 |
| LDH | 120-250 U/L | 622 | 725 | 579 | 287 |
| BNP | 0-100 pg/mL | 71 | - | - | - |
| ALT | 7-40 U/L | 42 | 49 | 64 | 17 |
| AST | 13-35 U/L | 78 | 96 | 91 | 26 |
| Albumin | 40-55 g/L | 27.3 | 31.4 | 32 | 41.1 |
| Complement C3 | 0.9-1.8 g/L | 0.787 | - | - | - |
| TSH | 0.27-4.20 mIU/L | - | 9.48 | - | 3.09 |
| FT3 | 3.1-6.8 pmol/L | - | 2.6 | - | 3.5 |
| FT4 | 12.0-22.0 pmol/L | - | 18.2 | - | 18 |

***Abbreviations*:** hs‑CRP, high‑sensitivity C‑reactive protein; ESR, erythrocyte sedimentation rate; ALT, alanine aminotransferase; AST, aspartate aminotransferase; LDH, lactate dehydrogenase; CK, creatine kinase; CK‑MB, creatine kinase‑myocardial band; Myo, myoglobin; hs‑cTnT, high‑sensitivity cardiac troponin T; BNP, B‑type natriuretic peptide; PCT, procalcitonin; TSH, thyroid‑stimulating hormone; FT3, free triiodothyronine; FT4, free thyroxine; C3, complement component 3; WBC, white blood cell count; Hb, hemoglobin.
