# Supplementary material for: Unilateral lower limb cellulitis-like edema as an atypical cutaneous manifestation of antisynthetase syndrome: a case report and targeted literature review
Source: Front Med (Lausanne). 2026 Sep 9;13:1942019. doi: 10.3389/fmed.2026.1942019 (PMC13597253; doi:10.3389/fmed.2026.1942019)
Supplement: Supplementary file 2 [file Supplementaryfile2.docx]

**Detailed clinical and serological scoring components of the CLASS classification criteria**

1. ILD with predominant NSIP and/or OP —15.3
2. Myositis assessed through biopsy —12.0
3. Inflammatory rashes not specific for DM —3.4
4. Antinuclear antibodies positive with cytoplasmic pattern —10.6
5. Anti-Jo1 positive (by any method) or non-anti-Jo1 positive by IP —21.7
6. Positive by any assay of anti-Ro52 or anti-SSA antibody —5.6

**Detailed scoring components of the 2017 EULAR/ACR classification criteria for adult and juvenile idiopathic inflammatory myopathies (with skin biopsy results)**

| **Variable** | **Score points (Biopsy)** |
| --- | --- |
| **Age of onset of first related symptoms** |  |
| 18–40 | 1.5 |
| ≥40 | 2.2 |
| **Muscle weakness** |  |
| Objective symmetric weakness, usually progressive, of proximal upper extremities | 0.7 |
| Objective symmetric weakness, usually progressive, of proximal lower extremities | 0.5 |
| Neck flexors are relatively weaker than neck extensors | 1.6 |
| In the legs, proximal muscles are relatively weaker than distal muscles | 1.2 |
| **Skin manifestations** |  |
| Heliotrope rash | 3.2 |
| Gottron's papules | 2.7 |
| Gottron's sign | 3.7 |
| **Other clinical manifestations** |  |
| Dysphagia or esophageal dysmotility | 0.6 |
| **Laboratory measurements** |  |
| Anti-Jo-1 (anti-histidyl-tRNA synthetase) autoantibody positivity | 3.8 |
| Elevated serum levels of creatine kinase (CK)* or lactate dehydrogenase (LDH)* or aspartate aminotransferase (ASAT/AST/SGOT)* or alanine aminotransferase (ALAT/ALT/SGPT)* | 1.4 |
| **Muscle biopsy features** |  |
| Endomysial infiltration of mononuclear cells surrounding, but not invading, myofibres | 1.7 |
| Perimysial and/or perivascular infiltration of mononuclear cells | 1.2 |
| Perifascicular atrophy | 1.9 |
| Rimmed vacuoles | 3.1 |
| *Total scores of this patient* | 9.1 |

***Note.*** IIM classification is determined by probability thresholds derived from statistical optimization and expert consensus: "definite IIM" requires ≥90% probability (score ≥7.5 without biopsy, ≥8.7 with), "probable IIM" ranges from ≥55% to <90% (score ≥5.5/6 without biopsy, ≥6.7 with), "possible IIM" spans ≥50% to <55%, and "non-IIM" is defined as <50% (score <5.3 without biopsy, <6.5 with). The patient achieved a total aggregate score of 9.1 (≥8.7), consistent with a diagnosis of "definite IIM"(≥90% probability). *Serum levels above the upper limit of normal.

**Detailed search strategy in PUBMED**

(("Antisynthetase Syndrome"[Mesh]) OR ("antisynthetase syndrome"[Title/Abstract]) OR ("antisynthetase"[Title/Abstract]) OR ("anti-Jo-1"[Title/Abstract]) OR ("anti-PL7"[Title/Abstract]) OR ("anti-PL12"[Title/Abstract]) OR ("anti-aminoacyl tRNA synthetase"[Title/Abstract])) AND ((edema[Title/Abstract]) OR (oedema[Title/Abstract]) OR (swelling[Title/Abstract]) OR ("limb swelling"[Title/Abstract]) OR (anasarca[Title/Abstract]) OR ("soft tissue swelling"[Title/Abstract]) OR (fasciitis[Title/Abstract]) OR ("cellulitis-like"[Title/Abstract]))
